# The Actual Cautery Successfully Employed in Gangrene of the Mouth

**Published:** 1845-08

**Authors:** 


*The Actual Cautery successfully Employed in Gangrene of the Mouth.*—The case occurred in a female child, five years of age, during the convalescence from an attack of Typhus fever. The peculiar

(by some deemed pathognomonic) offensive odour, that accompanies this species of gangrene, was remarked before any actual appearance of the disease was discoverable. There were four gangrenous patches on the upper and lower gums, besides one on the inner surface of the right cheek. Dr. Weber determined to apply the actual cautery to all the diseased spots, and the immediately adjacent parts. This was done, not without difficulty, as may be imagined; and the mouth was directed to be afterwards washed out every two hours with a decoction of Cinchona, to which some *spiritus cochlearecæ* was added. The offensive smell ceased immediately after the application of the cautery, and did not again return.

On the third day, the sloughy parts began to detach themselves; at the same time, a few suspicious-looking spots were touched with the nitrate of silver. Several teeth remained "*dechaussées*," and at three points the maxillary (upper) bone was exposed. These necrosed portions, inclosing a molar tooth, and two smaller bits of the lower jaw were subsequently detached. During the progress of the case, an offensive purulent discharge took place from the ears, and numerous small abscesses formed on different parts of the body.

Dr. Weber does not think that, in this case (nor indeed generally,) there was any reason to suspect that the gangrenous ulceration of the mouth was in any degree attributable to the action of mercury upon the system. He points out the analogy between this disease—which he designates by the name of *noma* or corroding ulcer—and the *pustule maligne*, so accurately described by Boyer and other continental writers.—*Ibid, from Gaz. Med. de Stras.*

